# Review of "Computational Modeling of Genetic and Biochemical Networks" edited by James M. Bower and Hamid Bolouri

**DOI:** 10.1186/1475-925X-4-56

**Published:** 2005-10-04

**Authors:** Werner Dubitzky

**Affiliations:** 1 School of Biomedical Sciences, University of Ulster, Coleraine, Co. Londonderry, BT52 1SA, UK

Since the coming-of-age of the scientific discipline called *bioinformatics *it has become increasingly clear that computers in biology will be important not only for managing and analyzing biological and biomedical data but also for modeling and simulation of life processes and systems. The field concerned with this aspect of computers in biology has become known as *computational biology*. Computational Modeling of Genetic and Biochemical Networks, edited by James M. Bower and Hamid Bolouri, is a text that deals exclusively with computational biology concepts and applications. The book can be seen as an advocate of this increasingly important discipline as it presents a range of problems and methodologies that demonstrate that biology can be approached systematically and systemically by fully characterizing – through a combination of theory, simulation and experiment – entire biological systems such as metabolism, signal transduction and gene regulation, their interacting biochemical networks, or even higher levels of biological organization. The book is organized into two parts: Part I. Modeling Genetic Networks and Part II. Modeling Biochemical Networks.

The first part of the book is concerned with models of gene regulation, including protein-DNA and DNA-DNA interactions. Five chapters are devoted to this topic, by reviewing several tools and methods that are currently available for unraveling genetic regulatory networks at various levels of resolution and abstraction. They include logical and probabilistic approaches, which are applied to problems including prokaryotic and eukaryotic systems. Chapter 1 provides an excellent introduction into the basics of gene regulation and reviews a range of methods and tools that have been used to model gene-regulatory mechanisms and systems. Against this background, Chapters 2 to 5 discuss different studies and methodologies in detail.

The second part of the book tackles protein interactions produced by gene regulation. It first considers interactions among few molecules and then goes on to present models aimed at understanding reactions and diffusion by large numbers of molecules. This part has six chapters. Although an overview chapter, like Chapter 1 in Part I is missing, collectively, the introductions of the six chapters provide an interesting and comprehensive overview of the different biological systems and their background and the relevant modeling methodologies and tools. The biological systems considered in this part include cell cycle regulation, signaling pathways, and excitable membranes and synaptic interactions.

Overall, the volume provides an excellent and broad overview of computational biology and the methodologies and tools needed to model and simulate complex biological systems. One message the book conveys is that tackling computational biology problems requires significant effort and considerable knowledge of mathematics, information technology (IT) and biology. While a great effort is made to cover the relevant background in biology, theory and IT, it could nevertheless be difficult to follow some of the detailed discussions. This is a consequence of presenting a wide range of biological problems and formal and computational methods.

